# Staphylococcal Protein A (*spa*) Locus Is a Hot Spot for Recombination and Horizontal Gene Transfer in Staphylococcus pseudintermedius

**DOI:** 10.1128/mSphere.00666-20

**Published:** 2020-10-28

**Authors:** Alem Zukancic, Mubin A. Khan, Sumayya J. Gurmen, Quinn M. Gliniecki, Dayna L. Moritz-Kinkade, Carol W. Maddox, Md Tauqeer Alam

**Affiliations:** a Department of Pathobiology, College of Veterinary Medicine, University of Illinois at Urbana-Champaign, Urbana, Illinois, USA; b School of Information Sciences, University of Illinois at Urbana-Champaign, Urbana, Illinois, USA; c Department of Veterinary Clinical Medicine, College of Veterinary Medicine, University of Illinois at Urbana-Champaign, Urbana, Illinois, USA; d Infection Genomics for One Health Theme, Carl R. Woese Institute for Genomic Biology, University of Illinois at Urbana-Champaign, Urbana, Illinois, USA; University of Nebraska Medical Center

**Keywords:** *Staphylococcus*, antibiotic resistance, genomics, horizontal gene transfer

## Abstract

Staphylococcus pseudintermedius is a major canine pathogen but also occasionally colonizes and infects humans. Multidrug-resistant methicillin-resistant S. pseudintermedius (MDR MRSP) strains have emerged globally, making treatment and control of this pathogen challenging. Sequence type 71 (ST71), ST68, and ST45 are the most widespread and successful MDR MRSP clones. The potential genetic factors underlying the clonal success of these and other predominant clones remain unknown. Characterization of the pangenome, lineage-associated accessory genes, and genes acquired through horizontal gene transfer from other bacteria is important for identifying such factors. Here, we analyzed genome sequence data from 622 S. pseudintermedius isolates to investigate the evolution of pathogenicity across lineages. We show that the predominant clones carry one or more lineage-associated virulence genes. The gene encoding staphylococcal protein A (SpA), a key virulence factor involved in immune evasion and a potential vaccine antigen, is deleted in 62% of isolates. Most importantly, we have discovered that the *spa* locus is a hot spot for recombination and horizontal gene transfer in S. pseudintermedius, where genes related to restriction modification, prophage immunity, mercury resistance, and nucleotide and carbohydrate metabolism have been acquired in different lineages. Our study also establishes that ST45 is composed of two distinct sublineages that differ in their accessory gene content and virulence potential. Collectively, this study reports several previously undetected lineage-associated genetic factors that may have a role in the clonal success of the major MDR MRSP clones. These data provide a framework for future experimental studies on S. pseudintermedius pathogenesis and for developing novel therapeutics against this pathogen.

**IMPORTANCE** Staphylococcus pseudintermedius is a major canine pathogen but can also occasionally infect humans. Identification of genetic factors contributing to the virulence and clonal success of multidrug-resistant S. pseudintermedius clones is critical for the development of therapeutics against this pathogen. Here, we characterized the genome sequences of a global collection of 622 S. pseudintermedius isolates. We show that all major clones, besides carrying core virulence genes, which are present in all strains, carry one or more lineage-specific genes. Many of these genes have been acquired from other bacterial species through a horizontal gene transfer mechanism. Importantly, we have discovered that the staphylococcal protein A gene (*spa*), a widely used marker for molecular typing of S. pseudintermedius strains and a potential vaccine candidate antigen, is deleted in 62% of strains. Furthermore, the *spa* locus in S. pseudintermedius acts as a reservoir to accumulate lineage-associated genes with adaptive functions.

## Abstract (precis)

Staphylococcus pseudintermedius is a major canine pathogen but can also occasionally infect humans. Identification of genetic factors contributing to the virulence and clonal success of multidrug-resistant S. pseudintermedius clones is critical for the development of therapeutics against this pathogen. Here, we characterized the genome sequences of a global collection of 622 S. pseudintermedius isolates. We show that all major clones, besides carrying core virulence genes, which are present in all strains, carry one or more lineage-specific genes. Many of these genes have been acquired from other bacterial species through a horizontal gene transfer mechanism. Importantly, we have discovered that the staphylococcal protein A gene (*spa*), a widely used marker for molecular typing of S. pseudintermedius strains and a potential vaccine candidate antigen, is deleted in 62% of strains. Furthermore, the *spa* locus in S. pseudintermedius acts as a reservoir to accumulate lineage-associated genes with adaptive functions.

## INTRODUCTION

Staphylococcus pseudintermedius is a leading cause of pyoderma, otitis externa, urinary tract infections, and postsurgical abscesses in dogs. It also occasionally infects humans, especially the pet owners and veterinarians in close contact with the infected animals. Almost all human cases of S. pseudintermedius infections are reported to be zoonotic transmissions from the infected companion animals, indicating that the same strain can infect both animals and humans (1–4). In humans, it has been associated with skin infections, ulcers, brain abscess, cardioverter defibrillator infection, bacteremia, cellulitis, endocarditis, and chronic rhinosinusitis (5–8). The emergence and global spread of multidrug-resistant methicillin-resistant S. pseudintermedius (MDR MRSP) strains poses a serious challenge for the treatment and control of this important pathogen (9–11). The pattern of the emergence and evolution of antibiotic resistance in S. pseudintermedius is very similar to the pattern observed in Staphylococcus aureus, with both species acquiring almost identical types of resistance against commonly used β-lactam and non-β-lactam antibiotics, including fluoroquinolones (9, 12). Zoonotic transmission of MDR MRSP strains also poses a high potential risk of horizontal transfer of antibiotic resistance genes to other bacterial species within the human host. It is worth noting that the *mecA* gene, carried by the staphylococcal cassette chromosome *mec* (SCC*mec*) element in methicillin-resistant S. aureus (MRSA), is thought to have been acquired from an animal-related commensal *Staphylococcus* species, Staphylococcus fleurettii, through horizontal gene transfer (HGT) (13). Horizontal transfer of the *mecA* locus from S. fleurettii converted methicillin-susceptible S. aureus (MSSA) into MRSA (13).

The population structure of S. pseudintermedius is heterogenous, with over 2,000 sequence types (STs) recorded to date in the PubMLST database (https://pubmlst.org/organisms/staphylococcus-pseudintermedius/). Some of these STs, such as ST71, ST68, and ST45, the predominant clones of Europe, North America, and Asia, respectively, have spread globally, becoming the most widespread MDR MRSP clones (9, 14). The emergence and clonal expansion of MDR MRSP clones ST496, ST181, and ST258 have also been observed in some countries (15, 16). The enhanced fitness and clonal success of the epidemic clones is generally associated with the acquisition of antibiotic resistance, mobile genetic elements, prophages, plasmids, and virulence factors that confer adaptive advantages that allow them to cause infections and spread successfully. For example, the clonal expansion of the virulent community-associated MRSA (CA-MRSA) clone USA300 occurred as a result of the acquisition of fluoroquinolone resistance (FQR) and the arginine catabolic mobile element (ACME) (12). The ACME carries genes for arginine catabolism, allowing the bacteria to survive at lower pHs on human skin. In a recent study, we identified four lineage-specific prophages in S. pseudintermedius (17). One of these prophages, SpST71A, carries putative antibiotic resistance and virulence genes and is inserted within the *comGA* gene of the genetic competence operon *comG* in the ST71 clone. The *comG* operon in all ST71 isolates is therefore disrupted and likely nonfunctional. The acquisition of SpST71A, together with *comG* disruption, may be contributing to the increased virulence and clonal success of the ST71 clone (17).

Similar to S. aureus, a large number of potential virulence factors have been identified in S. pseudintermedius, including surface proteins, exoenzymes, and exotoxins (18–21). Many of these virulence factors are encoded by lineage-associated accessory genes likely derived from other bacterial species through HGT. We hypothesize that the successful MDR MRSP clones harbor one or more of these lineage-associated genes that provide them adaptive advantages over other clones. In this study, we have characterized the genome sequences of 622 S. pseudintermedius isolates to identify all previously known genes that are likely involved in the virulence and clonal success of the major MDR MRSP clones. We have investigated all putative HGT-derived genes in these strains to gain insights on the lineage-specific acquisition of adaptive functions in the major lineages. A comprehensive analysis of the potential virulence factors and other lineage-associated HGT-derived genes is necessary to better understand the evolution of pathogenicity across different S. pseudintermedius lineages. The novel virulence genes identified through comparative genome approaches could serve as targets for therapeutic interventions.

## RESULTS AND DISCUSSION

### S. pseudintermedius harbors lineage-associated virulence genes.

**FIG 1:**
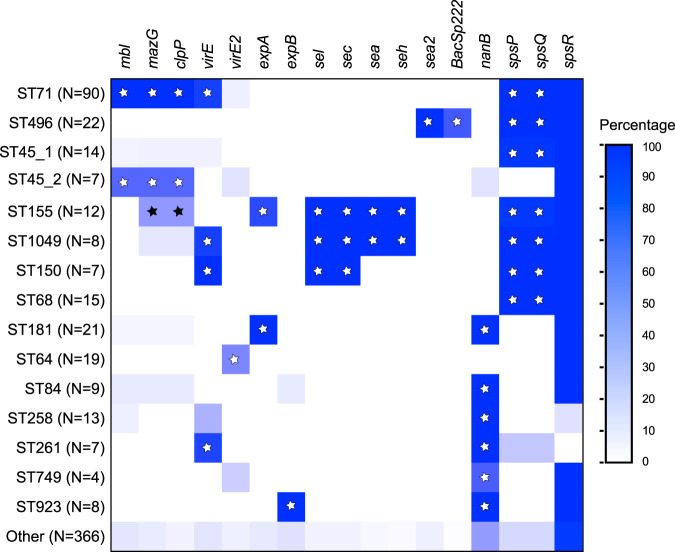
Heatmap showing the distribution of 17 noncore virulence genes. *mbl*, *mazG*, and *clpP* are located on the SpST71A prophage (17). *virE* (locus_tag *A9I65_10340*) and *virE2* (locus_tag *EW134_05725*) are almost identical in length, with ∼76% and ∼82% sequence identity at the nucleotide and amino acid levels, respectively. *sea* (locus_tag *EW134_02425*) and *sea2* (locus_tag *EW134_00155*) are identical in length, with ∼80% and ∼72% sequence identity at the nucleotide and amino acid levels, respectively. The *bacSp222* gene, associated with ST496, encodes a 50-amino-acid multifunctional peptide with both bacteriocin and virulence factor characteristics (62). Significant positive associations are indicated with white (*P* < 0.005) and black (*P* < 0.05) stars. N, number of isolates; ST45_1, ST45 sublineage 1; ST45_2, ST45 sublineage 2.

Whole-genome sequencing (WGS) has proven to be a powerful tool in studying the evolution of antibiotic resistance and virulence-associated factors in bacteria. WGS has provided an alternative and complementary high-throughput approach to identifying the underlying genetic factors responsible for clonal expansion of the hypervirulent epidemic clones. Here, we utilized whole-genome sequence data from 622 strains to gain insights on the evolution of pathogenicity and horizontal gene transfer across different S. pseudintermedius lineages. Fifty genomes were sequenced as part of this study, while the remaining 572 were retrieved from the NCBI RefSeq or SRA database (Table S1 in the supplemental material). The STs represented by 4 or more isolates were considered predominant and included ST71, ST496, ST45, ST155, ST1049, ST150, ST68, ST181, ST64, ST84, ST258, ST261, ST749, and ST923 (*n* = 256) (Fig. 1). The STs represented by 3 or fewer isolates were considered minor STs (*n* = 366) (Table S1). A total of 69 virulence-associated genes were interrogated in 622 genomes using the large-scale BLAST score ratio (LS-BSR) (Table S2). They belonged to five major functional groups: surface or cell wall-associated proteins, toxins, exoenzymes, proteases, and genetic regulators. Fifty-two of these genes were present in all 622 genomes, irrespective of their sequence types, indicating that they are part of the core genome. The expression levels of the core virulence genes, however, can vary in different lineages, leading to lineage-wise differences in pathogenicity. The levels of expression of virulence genes are just as important as their lineage-specific presence (22). The frequencies of the remaining 17 virulence-associated genes varied across lineages, and they formed the basis of our virulence gene content analysis (Fig. 1, Table S3).

10.1128/mSphere.00666-20.7 TABLE S1 Details of the 622 S. pseudintermedius genomes analyzed in this study. Download Table S1, XLSX file, 0.1 MB. Copyright © 2020 Zukancic et al. 2020 Zukancic et al. This content is distributed under the terms of the Creative Commons Attribution 4.0 International license.

10.1128/mSphere.00666-20.8 TABLE S2 Details of the 69 virulence-associated genes investigated in this study. Download Table S2, XLSX file, 0.01 MB. Copyright © 2020 Zukancic et al. 2020 Zukancic et al. This content is distributed under the terms of the Creative Commons Attribution 4.0 International license.

10.1128/mSphere.00666-20.9 TABLE S3 The *spa*-typing results and the LS-BSR analysis indicating presence or absence of the 17 noncore virulence genes. The antibiotic resistance genes identified in these isolates using ResFinder are also shown. Download Table S3, XLSX file, 0.1 MB. Copyright © 2020 Zukancic et al. 2020 Zukancic et al. This content is distributed under the terms of the Creative Commons Attribution 4.0 International license.

As shown in Fig. 1, each major lineage is characterized by a unique and specific set of virulence genes. For example, the virus-associated antigen E gene (*virE*), which is carried on a complete prophage, SpST71B, was found in ST71, ST1049, ST150, and ST261 (*P < *0.005). A novel homologue of *virE*, called *virE2*, was strongly associated with ST64 (*P < *0.005) (Fig. 1, Fig. S1). Four exfoliative toxin genes, *siet*, *speta*, *expA*, and *expB*, are reported in S. pseudintermedius (23–25). ExpA and ExpB toxins have been shown to digest canine desmoglein 1 (Dsg1) and cause intraepidermal splitting in canine skin (24, 25). While *siet* and *speta* genes were found in all 622 strains, *expA* was associated with ST155 and ST181 and *expB* was associated with ST923 (*P < *0.005) (Fig. 1). Similar distribution patterns of these four toxin-encoding genes have been reported in a previous study (26). The genes encoding staphylococcal enterotoxins (SEs) have been identified frequently in clinical isolates from dogs with cutaneous infections (27). The enterotoxin genes *sel* and *sec*, adjacent to each other in the genome, were associated with ST155, ST1049, and ST150 (*P < *0.005). The *sea* and *seh* enterotoxin genes, also adjacent to each other, were associated with ST155 and ST1049 (*P < *0.005). A novel variant of *sea*, called *sea2*, was identified in ST496 (Fig. 1, Fig. S2). The sialidase toxin gene *nanB* was associated with ST181, ST84, ST258, ST261, ST749, and ST923 (*P < *0.005). Sialidases are important enzymes for sugar acquisition in bacteria, and their role in disease pathogenesis has been shown in many bacterial pathogens (28). In Streptococcus pneumoniae, sialidases are involved in colonization and infection of the upper and lower murine respiratory tract (29, 30).

10.1128/mSphere.00666-20.1 FIG S1 Amino acid sequence alignment of VirE (locus_tag *A9I65_10340*) and VirE2 (locus_tag *EW134_05725*) virulence proteins. Download FIG S1, EPS file, 0.1 MB. Copyright © 2020 Zukancic et al. 2020 Zukancic et al. This content is distributed under the terms of the Creative Commons Attribution 4.0 International license.

10.1128/mSphere.00666-20.2 FIG S2 Amino acid sequence alignment of SeA (locus_tag *EW134_02425*) and the ST496-associated SeA2 (locus_tag *EW134_00155*) toxin proteins. Download FIG S2, EPS file, 0.1 MB. Copyright © 2020 Zukancic et al. 2020 Zukancic et al. This content is distributed under the terms of the Creative Commons Attribution 4.0 International license.

Whole-genome sequencing of S. pseudintermedius strain ED99 identified 18 *sps* genes (*spsA* to *spsR*) encoding putative cell wall-associated proteins (CWAPs) (18, 31). The exact functions of only some of these S. pseudintermedius surface proteins (Sps) have been deciphered to date. SpsD, SpsL, and SpsO have been shown to participate in bacterial attachment to the host extracellular matrix and, thus, are involved in the pathogenesis of S. pseudintermedius (32–34). Similarly, *spsQ*, which is analogous to the S. aureus staphylococcal protein A (SpA) gene (*spa*), has been reported to be an important virulence determinant in S. pseudintermedius (21, 31). SpsQ (SpA) protein expressed on the cell surface binds to the Fc region of canine IgG, thereby helping the bacteria to escape from opsonization and phagocytosis by the polymorphonuclear cells (21). SpsQ is also a widely used marker for molecular typing (*spa* typing) of S. pseudintermedius strains. Unlike in S. aureus, there are two orthologues of *spa* in S. pseudintermedius, *spsP* (*spa2*) and *spsQ* (*spa1*) (Fig. S3) (31). In ED99 and other *spa*-positive strains, *spsP* and *spsQ* genes are adjacent to each other in the *oriC* environ of the genome, where five other *sps* genes (*spsK*, *spsL*, *spsM*, *spsG*, and *spsJ*) are also located (31). We examined all 18 *sps* genes in our isolates and found that the frequencies of *spsP*, *spsQ* and *spsR* vary among STs (Fig. 1, Table S3). *spsP* and *spsQ* (*spa*) were strongly associated (*P < *0.005) with ST71, ST496, ST45 sublineage 1, ST155, ST1049, ST150, and ST68 (Fig. 1). Both *spsP* and *spsQ* were absent in ST45 sublineage 2, ST181, ST64, ST84, ST258, ST749, and ST923. The presence or absence of the *spsQ* (*spa*) gene was validated by PCR in 50 S. pseudintermedius isolates whose whole genomes were sequenced in this study (Fig. S4). Although previous studies have reported *spa* gene deletion in some S. pseudintermedius strains, we have for the first time established a clear association between *spa* gene deletion and genetic lineages (35, 36).

10.1128/mSphere.00666-20.3 FIG S3 EasyFig alignment of the same copies of the *spa* region showing homology between *spsP* (*spa2*) and *spsQ* (*spa1*) genes (X shape shown in light blue). *spsP* and *spsQ* genes share ∼75% and 72% sequence identity at the nucleotide and amino acid levels, respectively. The dark blue color indicates 100% identity between the two aligned regions. Download FIG S3, EPS file, 0.1 MB. Copyright © 2020 Zukancic et al. 2020 Zukancic et al. This content is distributed under the terms of the Creative Commons Attribution 4.0 International license.

10.1128/mSphere.00666-20.4 FIG S4 PCR validation of the *spa* gene in 50 isolates whole genome sequenced in this study. The 968-bp *spa* gene fragment encompassing the hypervariable X region was amplified using the newly designed primers spaF1 (5′-ACACCAAGTTTCGCAGAAGAAGGAG-3′) and spaR1 (5′-ACTGTTTCACCAGGTTGAACGACATG-3′) using the following PCR cycle parameters: 95°C for 2 min, then 25 cycles of 95°C for 30 s, 55°C for 30 s, 72°C for 1 min; and a final extension of 72°C for 10 min. As indicated, the *spa* gene did not amplify in ST45 sublineage 2 (ST45_2), ST64, and ST181, but it produced the expected band in ST71, ST150, and ST1049 isolates. The results of only 26 representative isolates are shown in this figure. Download FIG S4, EPS file, 0.1 MB. Copyright © 2020 Zukancic et al. 2020 Zukancic et al. This content is distributed under the terms of the Creative Commons Attribution 4.0 International license.

### A vaccine solely based on SpA may not be effective against all lineages.

Similar to S. aureus, the SpA antigen in S. pseudintermedius is being considered a potential vaccine candidate antigen (21, 37). However, unlike S. aureus, *spa* (*spsQ*) is not a core gene in S. pseudintermedius (18, 35). In our globally diverse data set, only 38% of isolates (239 of 622) were *spa* positive, while ∼62% of isolates (383 of 622) were *spa* negative, clearly indicating that a large number of strains do not harbor *spa* (Fig. 1, Table S3). It also indicates that *spa* gene function is not essential for bacterial survival, colonization, and establishment of infection. The loss of function resulting from *spa* gene deletion is likely compensated by cell wall-associated proteins encoded by other *sps* genes. It is important to note that the successful MDR MRSP clones ST71, ST68, ST496, and ST45 sublineage 2 have not lost the *spa* gene (Fig. 1). In an important study, Latronico et al. compared the *in vitro* adherence properties of four MRSP (2 ST71:*spa* positive, 1 ST68:*spa* positive, and 1 ST258:*spa* positive) and three MSSP (1 ST25:*spa* positive; 1 ST257:*spa* negative, and 1 ST259:*spa* negative) strains (38). The two *spa*-positive ST71 strains showed greater adherence to canine and human corneocytes than all other *spa*-positive and *spa*-negative non-ST71 MRSP/MSSP strains. The enhanced *in vitro* adherence of ST71 to corneocytes, however, was not linked to *spsQ* (*spa*), *spsP*, or any other CWAP genes examined in the study (38). As mentioned above, the *spa* gene is strongly associated with the genetic background of the strains (Fig. 1). We also examined whether *spa* gene presence is associated with methicillin resistance (*mecA* gene presence). Among 622 isolates analyzed, 323 were MRSP, while 299 were MSSP (Table S3). The *spa* positivity rates in MRSP and MSSP isolates were 56% (181 of 323) and 19% (58 of 299), respectively. Thus, a significant association lies between *spa* and methicillin resistance, where MRSP isolates are more likely to carry *spa* than MSSP isolates (*P < *0.001). As suggested in previous studies, our results solidify the inefficacy of *spa* typing as a useful method for differentiating all S. pseudintermedius strains (15, 16, 35, 39, 40). Instead, *spa* is a useful marker for molecular typing of strains belonging to certain genetic backgrounds. Moreover, a future vaccine consisting solely of SpA antigen may not provide protection against all S. pseudintermedius strains, as a large number of them are *spa* negative.

### The *spa* region is a hot spot for recombination and horizontal gene transfer.

**FIG 2:**
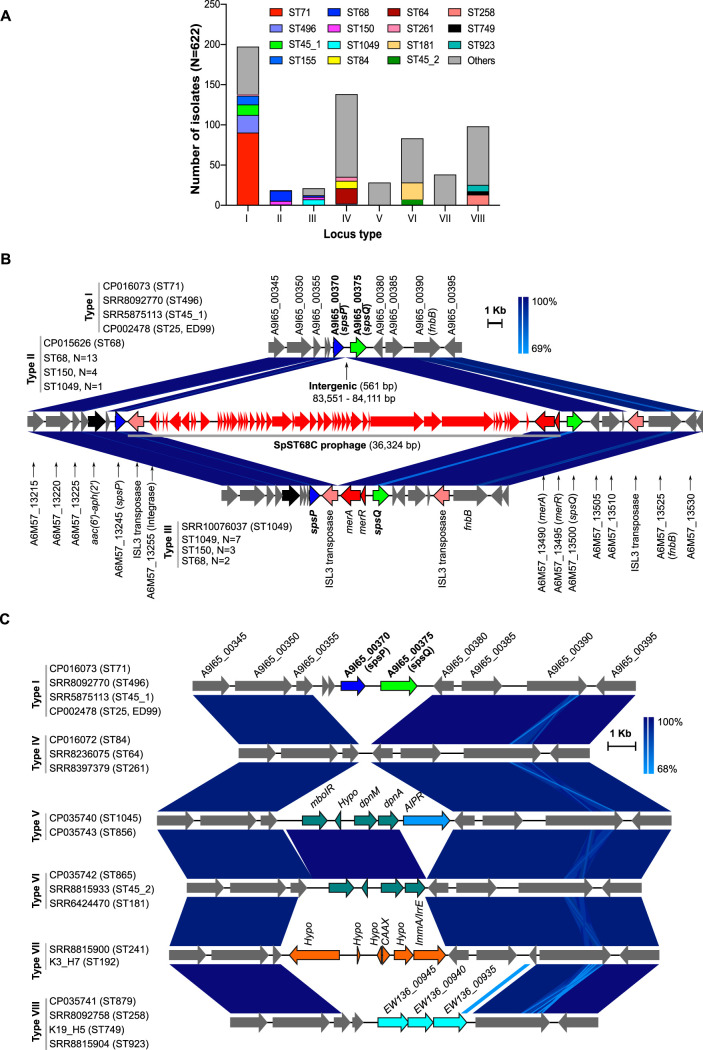
Acquisition of lineage-specific MGEs and accessory genes at the *spa* locus. (A) Eight types of *spa* loci (type I to type VIII) were identified in 622 S. pseudintermedius isolates. As shown, the *spa* region is conserved within a lineage (ST) but highly variable across lineages (STs). Major STs are represented by different colors, and all minor STs are shown grouped together in gray. (B) Types I, II, and III are *spa*-positive loci. In the type I locus (typified by the sequence with accession number CP016073 [CP016073 type]), *spsP* and *spsQ* genes are adjacent to each other, separated by a 561-bp intergenic region, whereas in the type II locus (CP015626 type), an intact prophage SpST68C (red ORFs), carrying mercury resistance genes *merA* and *merR*, is integrated between *spsP* and *spsQ*. In the type III locus (SRR10076037 type), the SpST68C prophage is deleted, while *merA* and *merR* genes are retained. The representative sequences of ST71 (CP016073), ST68 (CP015626) and ST1049 (SRR10076037) were used to create this linear comparison figure. (C) Type IV, V, VI, VII and VIII loci are *spa* negative. In the type IV locus (CP016072 type), both *spsP* and *spsQ* genes are deleted, but no new genes are integrated at that locus. In type V (CP035740 type) and type VI (CP035742 type) loci, a restriction modification (RM) system operon is integrated at the locus where *spa* was deleted. The RM operon in the type V locus also contains a putative abortive infection phage resistance (AIPR) protein. In the type VII locus (SRR8815900 type), a set of six genes (four hypothetical, one annotated as encoding CAAX protease self-immunity, and one annotated as encoding ImmA/IrrE family proteins) are integrated at the locus where *spa* was deleted. The type VIII locus (CP035741 type) contains three putative genes annotated as encoding NupC/NupG family nucleoside CNT transporter (locus_tag *EW136_00935*), a pseudouridine-5′-phosphate glycosidase (locus_tag *EW136_00940*), and a winged helix-turn-helix transcriptional regulator (locus_tag *EW136_00945*). The *spa*-positive (ST45_1) and *spa*-negative (ST45_2) sublineages of ST45 are also indicated.

Given that a large number of S. pseudintermedius strains do not carry *spa*, it was imperative to investigate the underlying mechanisms of its deletion. Comparative genomic analysis of the *spa* locus (*spsP* and *spsQ* combined) and the flanking sequences revealed that this region is highly variable across strains. The gene contents in this region vary significantly from one lineage to another, suggesting that the *spa* locus is one of the hot spots for recombination and genetic exchange in S. pseudintermedius. We classified this region into eight types (locus types I to VIII), based on the genes present in different lineages (Fig. 2A). Locus type I, as found in ST71, ST496, ST155, ST45 sublineage 1, and ST25, contained both *spsP* and *spsQ* genes, adjacent to each other, separated a 561-bp intergenic sequence (Fig. 2B). In ST68, ST150, and ST1049, a mobile genetic element (MGE) carrying putative mercury resistance genes *merA* and *merR* is integrated in the intergenic region between *spsP* and *spsQ* (type II and type III loci). The MGE in locus type II is a complete prophage that we have named SpST68C, as described previously (17). The MGE in locus type III consisted of only *merA* and *merR* genes flanked by ISL3 transposases (Fig. 2B). All *spa*-positive strains (*n* = 239), including minor STs, harbored one of these three locus types (Fig. 2A and B).

Analysis of the corresponding region in *spa*-negative strains (*n* = 383), where both *spsP* and *spsQ* were deleted, identified five different types of loci, types IV, V, VI, VII, and VIII (Fig. 2A and C). In locus type IV, both *spsP* and *spsQ* were deleted, but no new genes were found at the locus where *spa* was deleted. Locus type IV was also the most commonly found locus in minor STs (Fig. 2A). In locus types V and VI, restriction modification (RM) system genes *mboIR*, *dpnM*, and *dpnA* were acquired at the locus where *spa* was deleted (Fig. 2C). The RM system in locus type V had an additional gene that encodes a putative abortive infection phage resistance (AIPR) protein. The AIPR gene is most often found in RM system operons (41). In locus type VII, a set of genes with unknown functions were integrated at the locus where *spa* was deleted. One of these genes was annotated as encoding a CPBP family intramembrane metalloprotease with YdiL (CAAX protease, COG1266, functional category O) and Abi (CAAX protease self-immunity) domains. Abi (abortive infection) proteins are involved in self-immunity against cognate bacteriocin in some Gram-positive bacteria (42). They have also been shown to provide immunity against bacteriophages by interrupting their DNA replication, transcription, translation, maturation, and lysis steps (43, 44). In locus type VIII, a set of three genes, *EW136_00935* (nucleoside transporter protein, COG1972, functional category F), *EW136_00940* (pseudouridine-5′-phosphate glycosidase, COG2313, functional category F), and *EW136_00945* (winged helix-turn-helix transcriptional regulator), were integrated. Overall, the *spa*-negative strains have lineage-specific genes associated with prophage immunity, barriers to HGT, nucleotide transport, and metabolism at the locus where *spa* was deleted (Fig. 2C). The *spa* locus, therefore, acts as a reservoir where clones have acquired different sets of genes that may be associated with lineage-specific adaptive functions. Further characterization of these genes would be needed to determine their precise roles in S. pseudintermedius pathogenesis. These findings also suggest that the deletion of *spa* (*spsP* and *spsQ*) and the integration of new genes at that locus are two independent genetic events. As can be seen in locus type IV, *spa* is deleted but no new genes were acquired at that locus, indicating that *spa* deletion is an independent event. However, it is also possible that the genes were acquired at this locus but may have been lost by genetic drift or purifying selection (45, 46). In locus types II and III, MGEs are integrated without *spsP* and *spsQ* genes being deleted (Fig. 2B).

**FIG 3:**
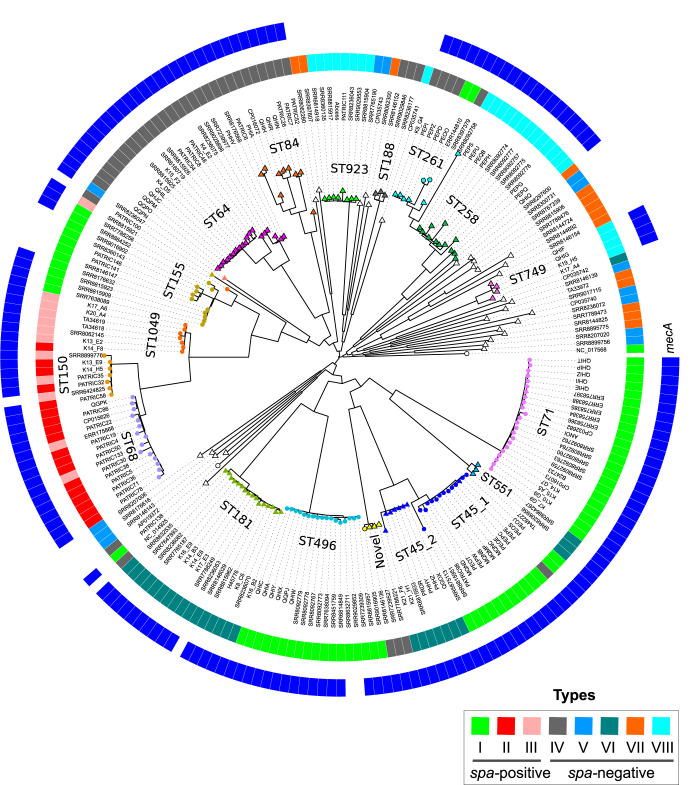
ML phylogeny showing *spa* locus types I to VIII in S. pseudintermedius lineages. Only 222 representative strains, including all major lineages and removing redundant STs, were used in this analysis. The genomes were aligned against the reference genome of S. pseudintermedius strain 081661 (CP016073), followed by recombination analysis by ClonalFrameML. The recombination-free alignment was used to infer an ML phylogeny using FastTree. Type I to VIII *spa* loci are shown in different colors as indicated. The branch tips labeled with circles are *spa*-positive isolates, while those marked with triangles are *spa*-negative isolates. The outer blue ring indicates *mecA* gene presence (MRSP) in the isolates.

The horizontal acquisition of genes with adaptive functions has played an important role in the evolution of bacterial pathogens (17, 47). Studies in other bacteria have shown that HGT usually occurs in specific locations, called “hot spots,” in the genome (45, 48, 49). Most MGEs and HGT-derived genes linked to antibiotic resistance, metal resistance, and virulence are integrated in the hot spot regions. Furthermore, the virulence genes involved in host cell attachment, invasion, and colonization show higher intensities of recombination and are most often located in the hot spot regions (49). For example, the *tbpA* and *tbpB* genes in Neisseria gonorrhoeae, Neisseria meningitidis, and Haemophilus influenzae, the *slpA* gene in Clostridium difficile, and the *clfA*, *sdrC*, *sdrD*, and *sdrE* genes in S. aureus are located in the recombination hot spot regions (49). Similar to the S. pseudintermedius *spsP* and *spsQ* genes, *tbpA* and *tbpB* in both *Neisseria* and *Haemophilus* are located adjacent to each other and encode surface proteins (49, 50). Collectively, the presence of *spsP* and *spsQ* orthologous genes adjacent to each other in the *oriC* environ and encoding surface proteins that are the target of natural selection makes them potential hot spots for recombination. The ClonalFrameML analysis conducted on a subset of 222 genomes verified the *spa* region as one of the hot spots of recombination in S. pseudintermedius (Fig. S5). Apart from ST45, almost all strains within a specific lineage had identical gene contents at the *spa* locus (Fig. 3). Among ST45 isolates, 13 isolates were *spa* positive with the type I locus, while 8 isolates were *spa* negative with the type VI locus. We refer to them as ST45 sublineage 1 (ST45_1) and ST45 sublineage 2 (ST45_2), respectively (Fig. 3).

10.1128/mSphere.00666-20.5 FIG S5 The recombination hotspots predicted by the ClonalFrameML. The ClonalFlameML analysis was performed to identify the putative recombination hotspots (shown in red) within the S. pseudintermedius genome. Only 222 representative samples were included in ClonalFrameML analysis. The *spa* region around the *oriC* environ is indicated. Download FIG S5, EPS file, 1.7 MB. Copyright © 2020 Zukancic et al. 2020 Zukancic et al. This content is distributed under the terms of the Creative Commons Attribution 4.0 International license.

### ST45 is composed of two distinct sublineages with different virulence potential.

**FIG 4:**
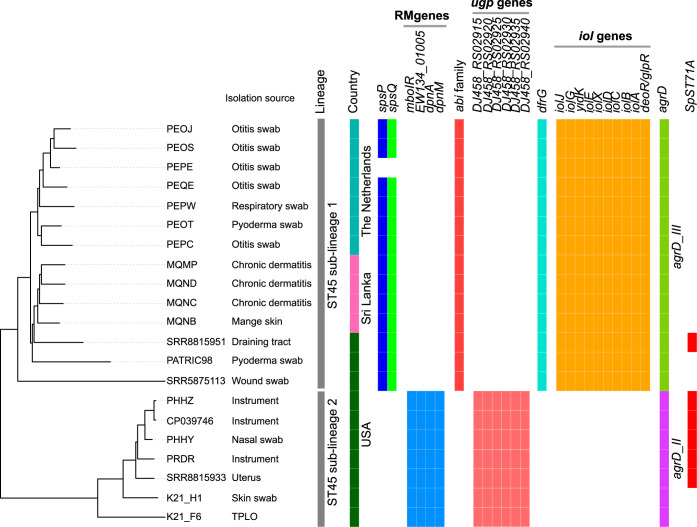
Whole-genome ML phylogeny and pan-GWAS analyses of the ST45 clone. The whole-genome ML phylogeny of the ST45 isolates was created using FastTree. The genes found to be enriched in *spa*-positive sublineage 1 and *spa*-negative sublineage 2 are shown along with the tree. As indicated, ST45 sublineage 1 carries *spsP*, *spsQ*, *dfrG*, *abi*, *iol*, and *agrD* type III genes. ST45 sublineage 2 has lost both *spsP* and *spsQ* genes and has acquired RM system genes *mboIR*, *EW134_01005*, *dpnA*, and *dpnM* at that locus. Similarly, they have lost the *abi* gene and have acquired a putative *ugp* operon (locus_tag *DJ458_RS02915*-*DJ458_RS02940*) at that locus. All isolates in sublineage 2 carry *agrD* type II, and five of them also harbor the SpST71A prophage (17). Similar to ST71, the SpST71A prophage is inserted within the *comG* operon of these five ST45 isolates, indicating that the prophage might have spread to ST45 from ST71 through HGT. The countries of origin and isolation sources of the isolates are also shown. *DJ458_RS02915* encodes a *sn*-glycerol-3-phosphate ABC transporter; *DJ458_RS02920* encodes a Gfo/Idh/MocA family oxidoreductase; DJ458_RS02925 encodes a carbohydrate ABC transporter permease; *DJ458_RS02930* encodes a sugar ABC transporter permease; *DJ458_RS02935* encodes a sugar ABC transporter substrate-binding protein; *DJ458_RS02940* encodes a helix-turn-helix domain-containing protein.

Twenty-one isolates in our data set were ST45, an MDR MRSP clone widely reported from Asia but also reported in the United States and Europe (36). The whole-genome phylogenetic analysis confirmed that ST45 is composed of two distinct sublineages (Fig. 4). ST45 sublineage 1 (ST45_1) contained isolates from Sri Lanka, The Netherlands, and the United States, whereas the sublineage 2 (ST45_2) isolates were all from the United States. To identify other genes that are significantly associated with these two sublineages, we performed pan-GWAS analysis of ST45 isolates using Scoary. The pangenome matrix of ST45 (3,127 total genes, 2,278 core genes, and 849 accessory genes) used for pan-GWAS analysis was constructed using Roary. Besides *spsP* and *spsQ*, several other genes were found to have a strong association (Benjamini-Hochberg-adjusted *P* value of <0.05) with sublineages 1 and 2 (Fig. 4). The RM genes *mboIR*, *dpnM*, and *dpnA* were only associated with sublineage 2, confirming the *spa* results described previously. Since these genes were always acquired at the locus where *spa* was deleted (type VI), no strain simultaneously carried both *spsP* and -*Q* and RM genes. Additionally, sublineage 2 harbored a putative *ugp* operon consisting of six genes (locus_tags *DJ458_RS02915*-*DJ458_ RS02940*) associated with *sn*-glycerol-3-phosphate and sugar transport (Fig. 4). The operon was integrated at a locus encoding a putative *abi* family protein in other S. pseudintermedius lineages. The *abi* gene and the *ugp* operon were never found simultaneously in any strain, suggesting that the acquisition of the *ugp* operon caused *abi* gene deletion. Pan-GWAS analysis also identified a putative *iol* operon, consisting of *depR-glpR*, *iolA*, *iolB*, *iolC*, *iolD*, *iolX*, *iolE*, *yidK*, *iolG*, and *iolJ* genes, associated with ST45 sublineage 1 (Fig. 4). The *iol* operon has been described in a number of bacterial species, including Bacillus subtilis, Salmonella enterica, Clostridium perfringens, Staphylococcus xylosus, and Lactobacillus casei (51–54). The bacteria harboring the *iol* operon are capable of utilizing *myo*-inositol (MI) as a carbohydrate source. In B. subtilis, the *iol* operon is comprised of *iolABCDEFGHIJ* genes, where *iolG* and *iolE* encode the enzymes required at the first and second steps of MI catabolism, respectively (51). It is likely that ST45 sublineage 1 utilizes MI as an alternative carbon and energy source to colonize and inhabit a particular ecological niche in the animal host or environment. It is important to note that the isolates containing the *iol* operon (ST45_1) were largely from dogs with clinical skin infections, while those with the *ugp* operon (ST45_2) were from medical instruments and opportunistic sites, such as skin and nasal passages, the uterus, and tibial-plateau-leveling osteotomy (TPLO) postoperative infection (Fig. 4). Future experimental studies would be required to establish the lineage-specific roles of *iol* and *ugp* genes in S. pseudintermedius pathogenesis. Although both sublineages were multidrug resistant, the *dfrG* gene responsible for trimethoprim resistance was only associated with sublineage 1 (Fig. 4, Table S3). Sublineages 1 and 2 also harbored different alleles of *agrD*, a well-characterized virulence-associated gene in *Staphylococcus*. In a previous study utilizing pulsed-field gel electrophoresis (PFGE), four different clusters were reported within ST45 (36). The cluster B isolates (*n* = 2 Thailand) were *spa* positive, while cluster C (*n* = 11 Thailand), D (*n* = 2 Thailand, *n* = 3 Israel), and E (*n* = 2 Thailand, *n* = 10 Israel) isolates were *spa* negative (36). Furthermore, in cluster B isolates, *spsP* and *spsQ* genes were adjacent to each other, similar to S. pseudintermedius strain ED99 (type I in this study), and in cluster C, D, and E isolates, RM genes were integrated at the locus where *spa* was deleted (type VI in this study). While both sublineages have been reported in the United States, only sublineage 1 has been reported in Europe (Fig. 4). Taken together, our results establish that ST45 is composed of two distinct sublineages, both of them having their origin in Asia. They carry different sets of genes related to virulence, prophage immunity, and metabolic pathways and, thus, may have differences in their virulence potentials. A large-scale phylogenetic and GWAS analysis of ST45 isolates from clinical infections and healthy dogs would help to better elucidate the evolution of virulence in these two sublineages.

**FIG 5:**
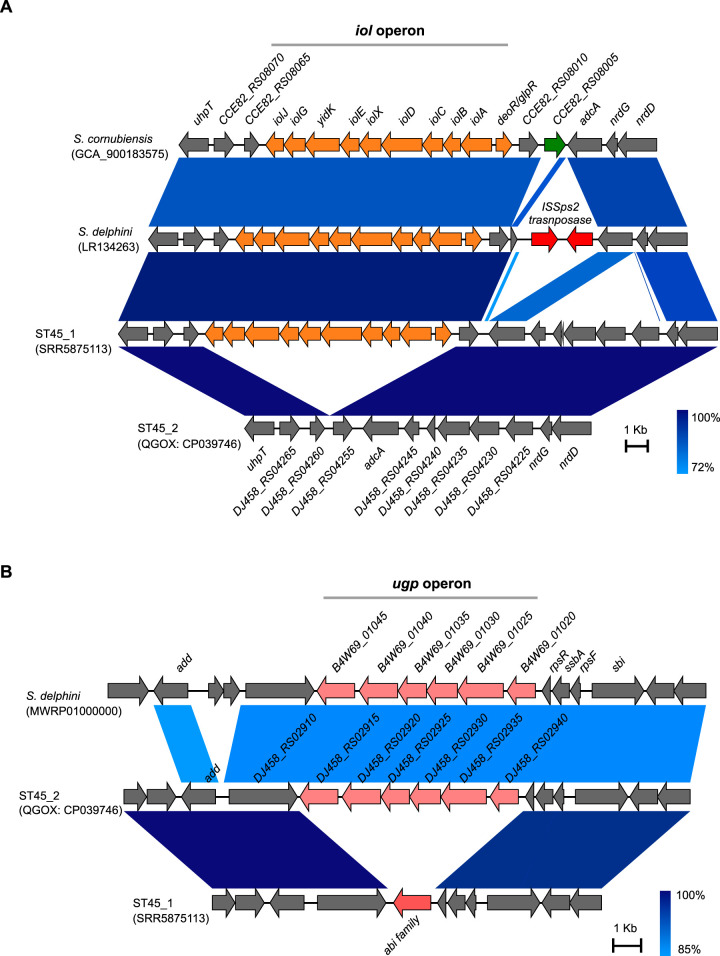
Genomic regions showing the putative *iol* and *ugp* operons in SIG. (A) Alignment showing the *iol* operon (orange ORFs) in S. cornubiensis, S. delphini, and S. pseudintermedius ST45_1. As indicated, the *iol* operon in S. delphini is in the vicinity of an insertion sequence (IS) element (ORFs in red). S. pseudintermedius ST45_2 does not have an *iol* operon between *DJ458_RS04255* and *DJ458_ RS04260*. (B) Alignment showing the putative *ugp* operon (pink ORFs) in S. delphini and S. pseudintermedius ST45_2. S. pseudintermedius ST45_1, on the other hand, has the intact *abi* gene. The accession numbers of the genome sequences used to create these figures are shown in parentheses.

The lineage-specific genes integrated at the *spa* locus and those identified from the pan-GWAS analysis of ST45 were further examined in all S. pseudintermedius genomes and other publicly available genomes of the Staphylococcus intermedius group (SIG) (Table S4). Members of the SIG include S. delphini, S. intermedius, S. pseudintermedius, and the newly identified novel species S. cornubiensis (20, 55, 56). We discovered that the *iol* and *ugp* operons were also present in all isolates belonging to ST181 (*n* = 21) and some other minor STs of S. pseudintermedius (Fig. S6). However, they were not identified in any isolates belonging to other major STs. Most significantly, 13 of the 22 S. delphini strains carried the *iol* operon, as does the only reported genome of S. cornubiensis (Fig. 5A, Fig. S6). One of the S. delphini genomes also carried the *ugp* operon (Fig. 5B, Fig. S6). The *iol* operon in S. delphini is located adjacent to a transposable element, suggesting that it may have been acquired from other bacteria through an HGT mechanism.

10.1128/mSphere.00666-20.6 FIG S6 Phylogenetic distribution of RM, *abi*, *ugp*, and *iol* genes across SIG. The S. pseudintermedius strains carrying one or more of these genes (*n* = 203) were combined with all publicly available S. delphini (*n* = 22) and S. intermedius (*n* = 6) genomes for this analysis. As indicated, ST45 sublineage 1 (SP ST45_1) carried *abi* and *iol* genes, whereas ST45 sublineage 2 (SP ST45_2) carried *ugp* (in place of *abi*) and RM (in place of *spa*) genes. Interestingly, all CC181 isolates were found to contain RM, *ugp* and *iol* genes. Many isolates belonging to minor STs also contained one or more of these genes. Thirteen of 22 S. delphini isolates carried *iol* genes, and one isolate each carried RM and *ugp* gene homologues. The *abi* gene was found in only one S. intermedius isolate. SP, S. pseudintermedius; SD, S. delphini; SI, S. intermedius. Download FIG S6, EPS file, 1.3 MB. Copyright © 2020 Zukancic et al. 2020 Zukancic et al. This content is distributed under the terms of the Creative Commons Attribution 4.0 International license.

10.1128/mSphere.00666-20.10 TABLE S4 Details of S. delphini, S. intermedius, and S. cornubiensis genomes analyzed in this study. Download Table S4, XLSX file, 0.01 MB. Copyright © 2020 Zukancic et al. 2020 Zukancic et al. This content is distributed under the terms of the Creative Commons Attribution 4.0 International license.

### The *iol* operon and genes acquired at *spa* and *abi* loci are putatively HGT derived.

**FIG 6:**
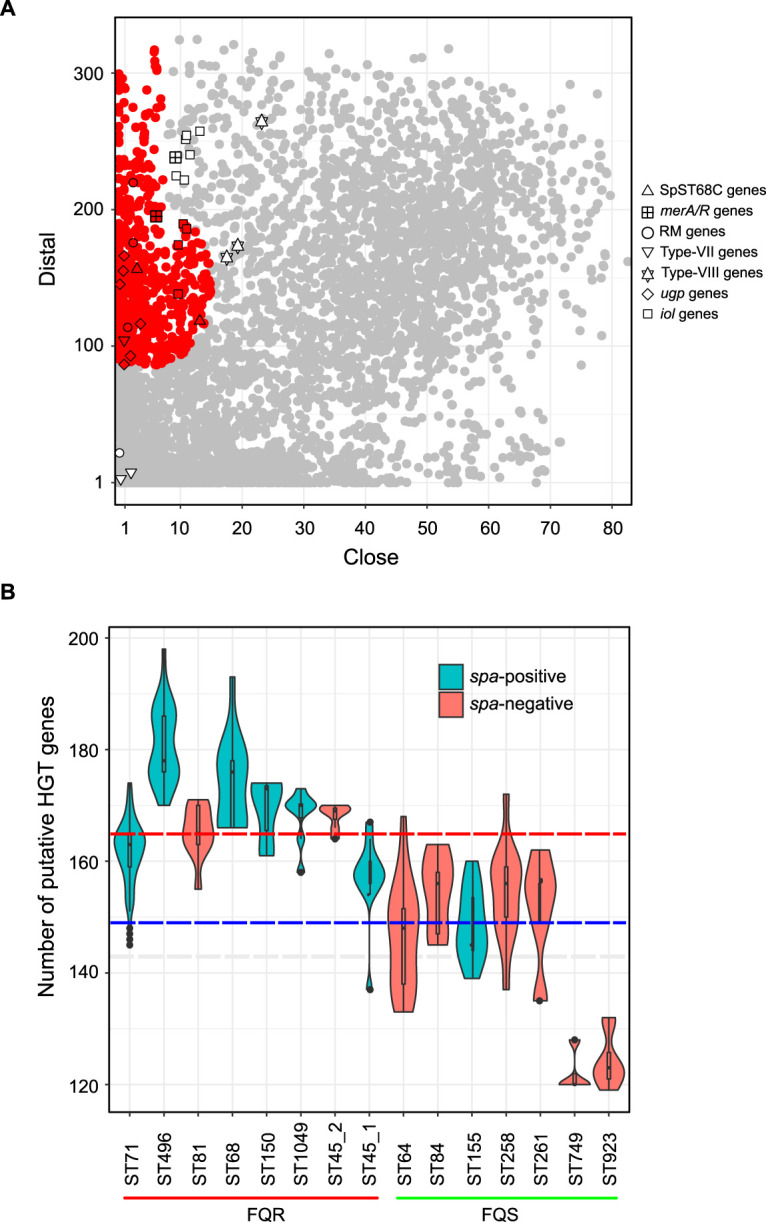
HGT-derived genes in S. pseudintermedius pangenome. (A) Scatterplot showing distal and close hits of all 10,370 unique genes (pangenome) from 622 S. pseudintermedius strains. The 771 predicted HGT-derived genes are shown in red, while vertically inherited genes (self-group) are shown in gray. As expected, genes associated with antibiotic resistance, virulence, restriction modification, and CRISPR-Cas and genes located on mobile genetic elements, prophages, and plasmids were predicted to be HGT-derived genes. The genes acquired at the *spa* locus and the *abi* locus and *iol* genes are indicated with different shapes. As shown, most of these genes were accurately predicted as HGT derived (within the red color zone), while some of them were close to the HGT group with high distal and low close scores. (B) Violin plot showing numbers of putative HGT-derived genes across S. pseudintermedius lineages. For this analysis, the HGT-derived genes were identified individually in each genome. FQR, fluoroquinolone-resistant clones with GyrA Ser84Leu and GrlA Ser80Ile mutations; FQS, fluoroquinolone-sensitive clones with wild-type GyrA and GrlA alleles. The dashed red and blue horizontal lines indicate the median numbers of HGT-derived genes in FQR (median = 165) and FQS (median = 148) clones, respectively.

To understand the role of horizontal gene transfer in S. pseudintermedius evolution, the putative HGT-derived genes were predicted in each strain (Fig. 6A). Of 10,370 genes estimated in the pangenome of 622 isolates, 771 (∼8%) were predicted to be putative HGT-derived genes by the HGTector. This included genes associated with antibiotic resistance [e.g., *blaZ*, *mecA*, *aac(6′)-aph(2″)*, *cat-(pC221)*, *dfrG*, *tet*(M), and *tet*(K)], plasmids, prophages, transposons, CRISPR-Cas, and RM systems. Most of the genes acquired at the *spa* locus (in types II, III, V, VI, VII, and VIII) were predicted to be HGT-derived genes. Similarly, the genes associated with the ST45_1 and ST45_2 sublineages were also classified as HGT-derived genes. The 771 HGT-derived genes belonged to a wide range of functional categories. A large number of them were linked to DNA replication, recombination, and repair, transcription, defense mechanisms, and basic metabolic functions. Approximately 20% of them were hypothetical and could not be assigned to any functional category by the eggNOG. We also examined the frequencies of putative HGT-derived genes among the major lineages. As shown in Fig. 6B, ST71, ST496, ST181, ST68, ST150, ST1049, and ST45 carry significantly higher numbers of HGT-derived genes than other clones in our data set. They also carry significantly higher numbers of antibiotic resistance determinants, including the GyrA and GrlA mutations conferring fluoroquinolone resistance (FQR) (17).

In conclusion, (i) the major S. pseudintermedius clones carry one or more lineage-associated virulence factors that are likely responsible for their pathogenicity and epidemiological success, (ii) *spa* is not a core gene in S. pseudintermedius, since it is deleted in ∼62% of strains, (iii) a vaccine based solely on the SpA antigen may not be effective against all strains, (iv) *spa* is a useful marker for molecular typing of only certain S. pseudintermedius lineages, (v) the *spa* locus is a hot spot for recombination and horizontal gene transfer, where genes associated with a wide range of functions have been acquired, (vi) ST45 consists of two distinct sublineages with different accessory gene repertoires and, possibly, virulence potential, and (vii) HGT has played an important role in the evolution of S. pseudintermedius clones. The lineage-associated genes identified in this study could serve as the basis for future studies on S. pseudintermedius pathogenesis and for developing novel therapeutics against this important pathogen.

## MATERIALS AND METHODS

### Whole-genome sequencing, assembly, and annotation.

Whole-genome sequence data of 622 S. pseudintermedius isolates representing all predominant STs were analyzed (Table S1 in the supplemental material). Fifty isolates were sequenced as part of this study as previously described (17). Briefly, genomic DNA was isolated from 1 ml of overnight culture grown in tryptic soy broth at 37°C, using the MasterPure Gram-positive DNA purification kit (Lucigen Corp., Middleton, WI). Paired-end library preparation and sequencing (2 × 150-bp paired-end reads, on the Illumina HiSeq 4000) was performed following the standard Illumina protocols (Illumina, Inc., San Diego, CA). Genome assembly was performed using SHOVILL, which utilizes the SPAdes version 2.5.0 assembler at its core (57, 58). The raw sequence reads have been submitted to the National Center for Biotechnology Information (NCBI)’s Sequence Read Archive (SRA) under BioProject accession number PRJNA564152. The remaining 572 were publicly available previously published genomes, retrieved as assemblies from NCBI’s RefSeq or as raw sequence reads from the SRA database. These raw reads were also assembled using SHOVILL. All genome assemblies were annotated using Prokka version 1.5.2 (59), and multilocus sequence types (MLSTs) were determined using mlst version 2.18.0 (https://github.com/tseemann/mlst).

### Screening of virulence genes.

We compiled a list of 69 virulence-associated genes from S. pseudintermedius and other *Staphylococcus* species (Table S2). This included genes involved in host adaptation, attachment and invasion, host cell lysis, immune evasion, biofilm formation, and regulation of the expression of genes involved in virulence (17, 21, 22, 26, 31, 32, 34, 55, 60–62). The presence of these genes in the genome assemblies of 622 strains was determined using large-scale BLAST score ratio (LS-BSR) with the *blastn* option (63). A gene was considered present with significant similarity if the BSR value was ≥0.80 and absent if the BSR was <0.40. A BSR value of 0.80 corresponds to ∼80% nucleotide identity over 80% of the sequence length (63). The assemblies were also scanned against the virulence factor database (VFDB) to look for additional virulence genes not included in our list (64). Fisher’s exact test was used to compare the prevalence of genes among different STs, and a two-sided *P* value of <0.05 was considered statistically significant.

### Analysis of the *spa* locus.

As described above, SpsQ (staphylococcal protein A [SpA]) constitutes an important virulence factor and a potential vaccine candidate antigen in S. pseudintermedius (21, 65, 66). Additionally, it is a widely used epidemiological marker for molecular typing (*spa* typing) of S. pseudintermedius strains. The LS-BSR analysis of the virulence genes described above revealed that the *spa* (*spsQ*) gene is absent in a large number of isolates. We validated the LS-BSR results by alternate methods. We performed *in silico* PCR on all genome assemblies with the widely used *spa*-typing primers SIspaF and SIspaR (14, 35, 36, 67). However, our analysis of the complete *spa* gene sequence alignment from over 200 isolates indicated that the gene in some isolates has polymorphisms in the primer-binding sites and, thus, may not have amplified using the above-named primers. Thus, new primers spaF1 (5′-ACACCAAGTTTCGCAGAAGAAGGAG-3′) and spaR1 (5′-ACTGTTTCACCAGGTTGAACGACATG-3′) were designed from the highly conserved regions, and another round of *in silico* PCR was performed. The *spa* gene was also verified by *spa* typing of the genomes using *get_spa_type*, a script originally developed for *spa* typing of S. aureus (https://github.com/mjsull/spa_typing). The repeat sequences and repeat orders used in this script to call *spa* types were obtained from the Pse-Spa database (http://www.pse-spa.org) maintained by Arshnee Moodley.

### Analysis of recombination and genetic exchange at the *spa* locus.

The *spa*-typeability rate in S. aureus is 100%, as the gene is present in all strains, irrespective of their sequence types (68). In contrast, our meta-analysis of the published studies indicated that the *spa*-typeability rate in S. pseudintermedius is ∼58%, with studies reporting typeability rates as low as 15 to 20%. Deletion of the *spa* gene in certain S. pseudintermedius strains has been reported previously (31, 35). However, no study has investigated the association of *spa* gene deletion with S. pseudintermedius genetic lineages. The location of the *spa* gene in the *oriC* region adjacent to an orthologous gene, extensive single nucleotide and repeat polymorphisms, and its role in host-pathogen interaction make it a potential hot spot for recombination. The region around *oriC* in S. aureus has been shown to exhibit significantly higher rates of recombination than other regions in the genome (48). We used ClonalFrameML on a select set of 222 genomes, including all major STs, to identify the genome-wide hot spots of recombination in S. pseudintermedius (69). The whole-genome alignment used in ClonalFrameML was produced by aligning the genome assemblies against the reference genome of S. pseudintermedius strain 081661, using Scapper (https://github.com/tseemann/scapper). The maximum-likelihood (ML) phylogeny was inferred from the putative recombination-free alignment using FastTree version 2.1.10 (70). To assess the extent of deletion in the *spa* region, we interrogated the *spa* locus and its flanking regions (6,832 bp upstream and 7,944 bp downstream) in all 622 strains using EasyFig (71). The open reading frames (ORFs) within this region were annotated using eggNOG to get insights on their biological functions (72).

### Phylogenetic and pan-GWAS analyses of ST45.

We found that only 13 of the 21 ST45 isolates harbored *spa* genes, indicating that there might be two sublineages within ST45 (36). To validate this result, we performed whole-genome-based ML phylogeny and pangenome-wide association (pan-GWAS) analysis of all 21 ST45 isolates (Table S1). Pan-GWAS analysis was performed with Scoary version 1.6.16 (73) on the pangenome matrix generated using Roary version 3.6.8 (17, 74). Scoary was developed to identify the accessory gene(s) associated with a particular phenotypic trait, such as antibiotic resistance, virulence, and group membership. We used the absence (denoted as 0) and presence (denoted as 1) of the *spa* gene as binary traits in Scoary to identify which other genes were enriched in *spa*-positive and *spa*-negative ST45 isolates. Only genes with a Benjamini-Hochberg-adjusted *P* value of <0.05 were considered associated with the presence or absence of *spa*. The genes identified through pan-GWAS were also interrogated in all non-ST45 S. pseudintermedius genomes and the publicly available genomes of S. delphini, S. intermedius, and S. cornubiensis (Table S4).

### Detection of HGT-derived genes in S. pseudintermedius.

Genes putatively acquired though horizontal gene transfer (HGT) were identified using HGTector version 2.0b1 (75). The reference pangenome consisting of 10,370 gene clusters predicted from 622 S. pseudintermedius strains by Roary, as described above, was used as the input for HGTector. A protein sequence similarity search was performed using Diamond version 0.9.31 (76) against the NCBI nonredundant (nr) database, with minimum search cutoffs set at an E value of 1e−50, sequence identity of 80%, and percentage query coverage of 80%. The database was created using the default *database* command of HGTector (on 25 March 2020), which downloads, per species, one representative proteome of all microorganisms available in the NCBI RefSeq database. S. pseudintermedius (NCBI TaxID 283734) was set as the self group, and genus *Staphylococcus* (NCBI TaxID 1279) as the close group. HGTector analyzes the search results of the proteins and clusters them into the self, close, or distal group according to their NCBI taxonomy. It also calculates a Silhouette score (range of −1 to 1, with higher values being better) for each gene, a metric which measures the confidence level of a data point assigned to the current cluster in the whole data set. Based on the clustering of “close” and “distal” scores, the program predicts genes that are susceptible to HGT. Genes with low close and high distal scores are considered putative HGT-derived genes.

### Data availability.

The raw sequence reads of the 50 isolates sequenced as part of this study have been submitted to the NCBI SRA under BioProject accession number PRJNA564152. Accession numbers of all genome sequences used in this study are listed in Table S1.
